# Age-related differences in drug-induced liver injury: a retrospective single-center study from a large liver disease specialty hospital in China, 2002–2022

**DOI:** 10.1007/s12072-024-10679-1

**Published:** 2024-06-19

**Authors:** Simiao Yu, Jiahui Li, Tingting He, Haocheng Zheng, Sici Wang, Yongqiang Sun, Liping Wang, Jing Jing, Ruilin Wang

**Affiliations:** 1 Department of Hepatology and Traditional Chinese Medicine, The Fifth Medical Center, PLA General Hospital, 100 West Fourth Ring Middle Road, Fengtai District, Beijing, 100039 China; 2 School of Traditional Chinese Medicine, Beijing University of Chinese Medicine, Beijing, 100029 China

**Keywords:** Hepatotoxicity, Drug-induced liver injury, Aging, Pediatric, Epidemiology, Medication, Age differences, Pharmacovigilance, Adverse drug reactions, Children, Geriatric

## Abstract

**Background and aims:**

Drug-induced liver injury (DILI) is a prevalent adverse reaction in clinical settings. However, there is limited research on age-related differences in DILI. We performed a large-scale retrospective study to delineate the characteristics of DILI across different age groups.

**Methods:**

We collected data on a total of 17,946 patients with confirmed DILI hospitalized at the Fifth Medical Center of the People’s Liberation Army (PLA) General Hospital in Beijing, China, from January 1, 2002, to December 31, 2022. The patients were stratified based on age into the following groups: children (< 18 years), young adults (18–44 years), middle-aged individuals (45–64 years), and elderly individuals (≥ 65 years). We gathered demographic information, medical histories, laboratory results, disease severity assessments, and mortality statistics for all patients.

**Results:**

Overall, the distribution of DILI cases across different age groups was as follows: 6.57% were children, 24.82% were young adults, 49.06% were middle-aged individuals, and 19.54% were elderly individuals. The percentage of females increased with age, rising from 36.47% in the pediatric group to 60.51% in the elderly group. Notably, central nervous system agents (15.44%) and anti-infectious agents (21.80%) were more commonly associated with DILI in children, while cardiovascular agents (10.58%) and herbal dietary supplements or traditional medicines (H/TMs) (26.29%) were more prevalent among elderly people with DILI. Among all age groups, hepatocellular-type DILI was more common in the pediatric group (*p* < 0.001), whereas cholestatic-type DILI and chronic DILI were more prevalent in the elderly group (*p* < 0.001). Acute liver failure (ALF) and fatal outcomes were more prevalent in the pediatric and elderly groups, particularly in the pediatric group (2.04%, *p* = 0.041; 0.85%, *p* = 0.007, respectively).

**Conclusions:**

Children and elderly individuals face a higher risk of adverse outcomes following DILI.

**Graphical Abstract:**

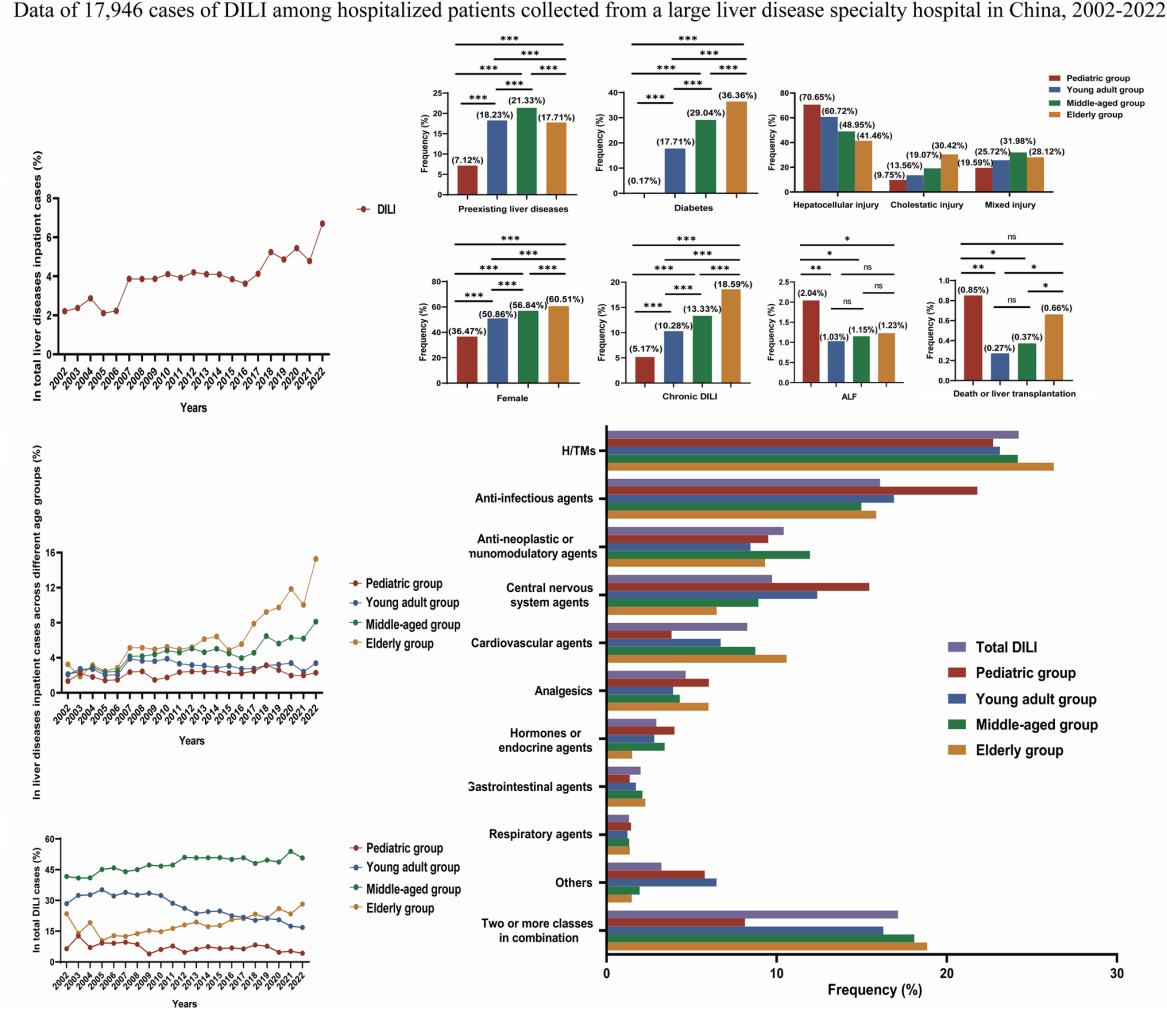

**Supplementary Information:**

The online version contains supplementary material available at 10.1007/s12072-024-10679-1.

## Introduction

Drug-induced liver injury (DILI) is one of the most common adverse drug reactions leading to acute liver failure and even death [1]. DILI is increasingly recognized as one of the most challenging diseases to treat in the clinic. With the continuous emergence of various new drugs, the diversity and quantity of pharmaceuticals have gradually increased, as has the widespread use of various herbal and dietary supplements, resulting in an upward trend in the incidence of DILI [2]. In Western countries, the annual incidence rate of DILI is estimated to range from approximately 1 in 100,000 to 20 in 100,000 individuals [3]. A retrospective study utilizing the UK-based General Practice Research Database revealed an annual incidence rate of DILI of 2.4 cases per 100,000 people [4]. Moreover, a population-based prospective study conducted in Iceland reported an annual incidence rate of 19 cases per 100,000 residents [5]. In a survey conducted by gastroenterology specialists in Delaware, USA, focusing on suspected DILI patients, an annual incidence of 2.7 cases per 100,000 adults was observed [6]. The incidence of DILI in China has shown an upward trend annually [7]. According to a large-scale retrospective study in the Chinese population, the incidence rate of DILI in mainland China is at least 23.8 per 100,000 individuals, substantially exceeding the figures reported in Western countries [8].

Age plays a major role in drug absorption, distribution, metabolism, and excretion [9, 10]. In comparison to adults, pediatric populations exhibit notable differences in phase I enzymes (oxidative enzymes such as cytochrome P450 CYP3A7, CYP3A4, and CYP1A2, as well as reductases and hydrolases) and phase II enzymes (such as N-methyltransferases and glucuronyl transferases) [11]. Conversely, as individuals age, their liver mass, regenerative capacity, and hepatic blood flow decrease, leading to a gradual decline in the activity of drug-metabolizing enzymes and the first-pass clearance rate of specific drugs [12]. Age-related physiological changes may considerably impact the ability to metabolize drugs, creating differences in drug elimination between children and elderly individuals [13]. To effectively mitigate the unpredictable risk of DILI in children and elderly individuals, there is an urgent need for epidemiological studies on the characteristics of DILI across different age groups. This will provide a better understanding of the specificity and manifestations of DILI at different ages. Unfortunately, research on age-related differences in DILI is limited [13]. Therefore, we conducted a large-scale retrospective study to delineate the characteristics of DILI in hospitalized patients in various age groups, encompassing implicated drugs and their clinical features, while also comparing the differences in age as a contributing factor to DILI.

## Methods

### Study design and data collection

This was a single-center retrospective study conducted at the Fifth Medical Center of the People’s Liberation Army (PLA) General Hospital in Beijing, China. We collected and analyzed data from all patients admitted with DILI between January 1, 2002, and December 31, 2022. Our focus was on discharge diagnoses that encompassed terms such as “drug-induced liver injury,” “drug-induced hepatitis,” “drug-induced cirrhosis,” “drug-induced liver failure,” or any other diagnostic terminology suggestive of liver injuries likely triggered by pharmaceuticals. Moreover, patients with preexisting liver diseases who developed DILI were considered eligible if the discharge diagnoses confirmed a DILI event. We excluded reports that were categorized as either “excluded (score ≤ 0)” or “unlikely (1–2)” by the Roussel–Uclaf Causality Assessment Method (RUCAM), including those reports with only causality judgments of “possible (3–5),” “probable (6–8),” or “highly probable (> 8) [14]. Detailed data, including demographic information, medical histories, information regarding the suspected drug causing liver injury, clinical features, laboratory test results, and histological findings, were extracted from the electronic medical records of all eligible patients. Each patient was assigned a unique ID in our electronic medical records, enabling the identification of multiple visits or readmissions and thus preventing duplication.

The study protocol was reviewed and approved by the Ethics Committee of the Fifth Medical Center of the PLA General Hospital in Beijing, China (KY-2022-1-3-1). Due to the retrospective nature of the analysis of existing administrative and clinical data, the requirement for obtaining informed consent from patients was waived by the Ethics Committee.

## Clinical types and severity of DILI

DILI types were classified as hepatocellular, cholestatic, or mixed based on the R ratio, which was calculated based on liver test results obtained at the time of presentation (R value = serum [alanine transaminase (ALT)/ALT upper limits of normal (ULN)]/[alkaline phosphatase (ALP)/ALP ULN]). DILI was classified as hepatocellular type when the R value was ≥ 5.0, cholestatic type when the R value was ≤ 2.0, and mixed type when the R value fell within the range of 2.0 to 5.0 [7]. Chronic DILI was defined as the presence of ongoing liver injury, substantial histological evidence of fibrosis, or even cirrhosis that persisted 6 months after the onset of DILI [7]. The definition of acute liver failure (ALF) comprises the presence of coagulation abnormalities, as indicated by an international normalized ratio (INR) of ≥ 2.0, hepatic encephalopathy symptoms, and total bilirubin levels ≥ 10 times the ULN, which equates to 10 mg/dL or 171 μmol/L, or consecutive daily increases of ≥ 1.0 mg/dL (17.1 μmol/L) within an illness duration of less than 26 weeks[15].

## Statistical analysis

We performed the data analysis using the Statistical Package for the Social Sciences (SPSS) 22.0 for Windows (SPSS, Chicago, Illinois, USA). Values are presented as medians with interquartile ranges or as percentages, as applicable. The incidence of DILI was determined by annually calculating the number of inpatient cases of DILI and dividing it by the total number of inpatient cases for liver disease. Between-group differences were assessed using either the Mann‒Whitney U test or Kruskal‒Wallis test for continuous variables. Categorical variables were analyzed using the χ^2^ test, Cochran-Mantel–Haenszel (CMH)-χ^2^ test, or Fisher’s exact test, as appropriate. Two-sided 95% confidence intervals were calculated, and statistical tests were considered significant at a two-sided alpha level of 5%.

## Results

### Demographic and clinical features

In this study, we gathered a total of 19,628 cases of DILI among hospitalized patients from the Fifth Medical Center of PLA General Hospital between January 1, 2002, and December 31, 2022. Of the initial 19,628 patients initially diagnosed with DILI at discharge, 1,682 patients with missing data were excluded. Consequently, the dataset eligible for analysis comprised 17,946 patients.

**Fig. 1:**
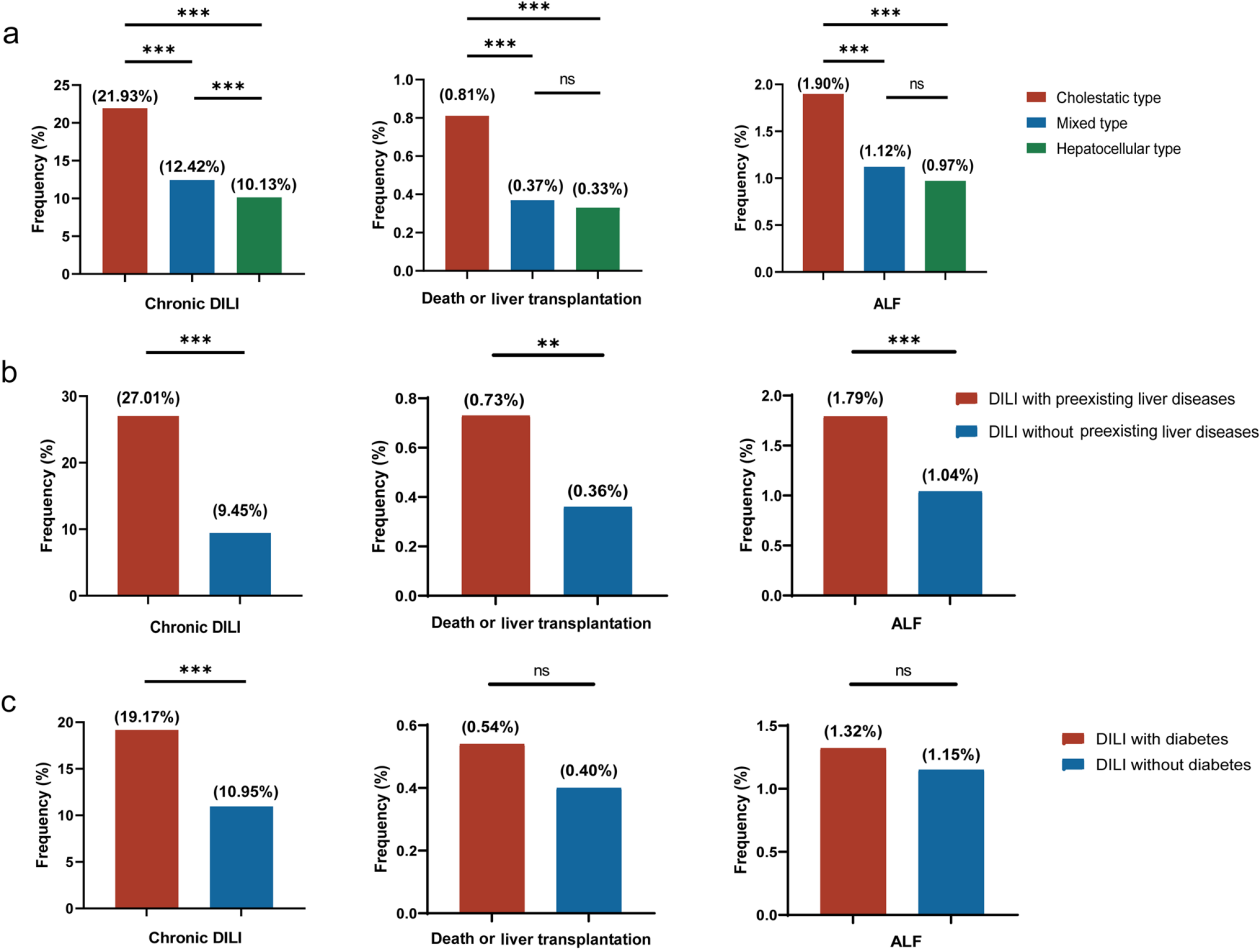
Effects of preexisting liver diseases and different clinical types of DILI on the prognosis of DILI patients; **a** Comparison of DILI prognosis among patients with different clinical types of DILI; **b** Comparison of DILI prognosis among patients with and without preexisting liver diseases; **c** Comparison of DILI prognosis among patients with and without diabetes. Note, *p < 0.05; **p < 0.01; ***p < 0.001; ns, not significant

As shown in Table 1, these patients were stratified by age into the following groups: the pediatric group (< 18 years), the young adult group (18–44 years), the middle-aged group (45–64 years), and the elderly group (≥ 65 years). The highest proportion of DILI cases was observed in middle-aged individuals (49.06%), followed by young adults (24.82%), elderly individuals (19.54%), and children (6.57%). Notably, Female (54.74%) were slightly more commonly affected by DILI than Male were (45.26%). The majority of DILI patients were of Han Chinese ethnicity, which is consistent with the overall population composition. Moreover, the majority of DILI patients presented with the hepatocellular type (51.83%), followed by the mixed type (28.86%) and cholestatic type (19.31%) (Table 1). Notably, greater proportions of cholestatic DILI were found in patients with chronic DILI (21.93%, *p* < 0.001) and ALF (1.90%, *p* < 0.001) and in patients who died or underwent liver transplantation (0.81%, *p* = 0.001) (Fig. 1a) (Supplementary Table 1). Out of the total patients, 15,601 (86.93%) were diagnosed with acute DILI, while 2,345 (13.07%) progressed to chronic DILI, characterized by persistent evidence of liver injury for at least 6 months after the onset of DILI. Notably, a limited number of patients progressed to severe outcomes, with 214 (1.19%) progressing to ALF, 2 (0.01%) undergoing liver transplantation, and 76 (0.42%) dying. Interestingly, we noted a substantial portion within our cohort, comprising 3695 (20.59%) individuals with DILI with preexisting liver diseases and 4623 (25.76%) DILI patients with diabetes (Table 1). Additionally, our study revealed that individuals with preexisting liver diseases were at an increased risk of chronic DILI (27.01% vs. 9.45%), ALF (1.79% vs. 1.04%), and mortality or requiring liver transplantation (0.73% vs. 0.36%) compared to those without preexisting liver diseases (Fig. 1b) (Supplementary Table 2). Diabetes was associated with increased chronicity (19.17% vs. 10.95%) in cases of DILI (Fig. 1c) (Supplementary Table 3).

**Table 1:** Demographic and clinical features of 17,946 DILI patients

	Number	%	95% CI
Sex			
Male	8123	45.26	[44.54–45.99]
Female	9823	54.74	[54.01–55.46]
Age (years)			
Children (< 18)	1179	6.57	[6.21–6.93]
Young adults (18–44)	4455	24.82	[24.19–25.46]
Middle-aged individuals (45–64)	8805	49.06	[48.33–49.80]
Elderly individuals (≥ 65)	3507	19.54	[18.96–20.12]
Ethnicity			
Han	17,126	95.43	[95.13–95.74]
Non-Han	820	4.57	[4.26–4.87]
Diabetes			
Yes	4623	25.76	[25.12–26.40]
No	13,323	74.24	[73.60–74.88]
Preexisting liver diseases			
Yes	3695	20.59	[20.00–21.18]
No	14,251	79.41	[78.82–80.00]
Clinical types of DILI			
Hepatocellular type (R ≥ 5)	9302	51.83	[51.10–52.56]
Cholestatic type (R ≤ 2)	3465	19.31	[18.73–19.89]
Mixed type (2 < R < 5)	5179	28.86	[28.20–29.52]
Acute/chronic DILI			
Acute DILI	15,601	86.93	[86.44–87.43]
Chronic DILI	2345	13.07	[12.57–13.56]
Severe outcomes			
Progression to ALF	214	1.19	[1.03–1.35]
Liver transplantation	2	0.01	[0.00–0.03]
Death	76	0.42	[0.33–0.52]

## Effect of age

**Fig. 2:**
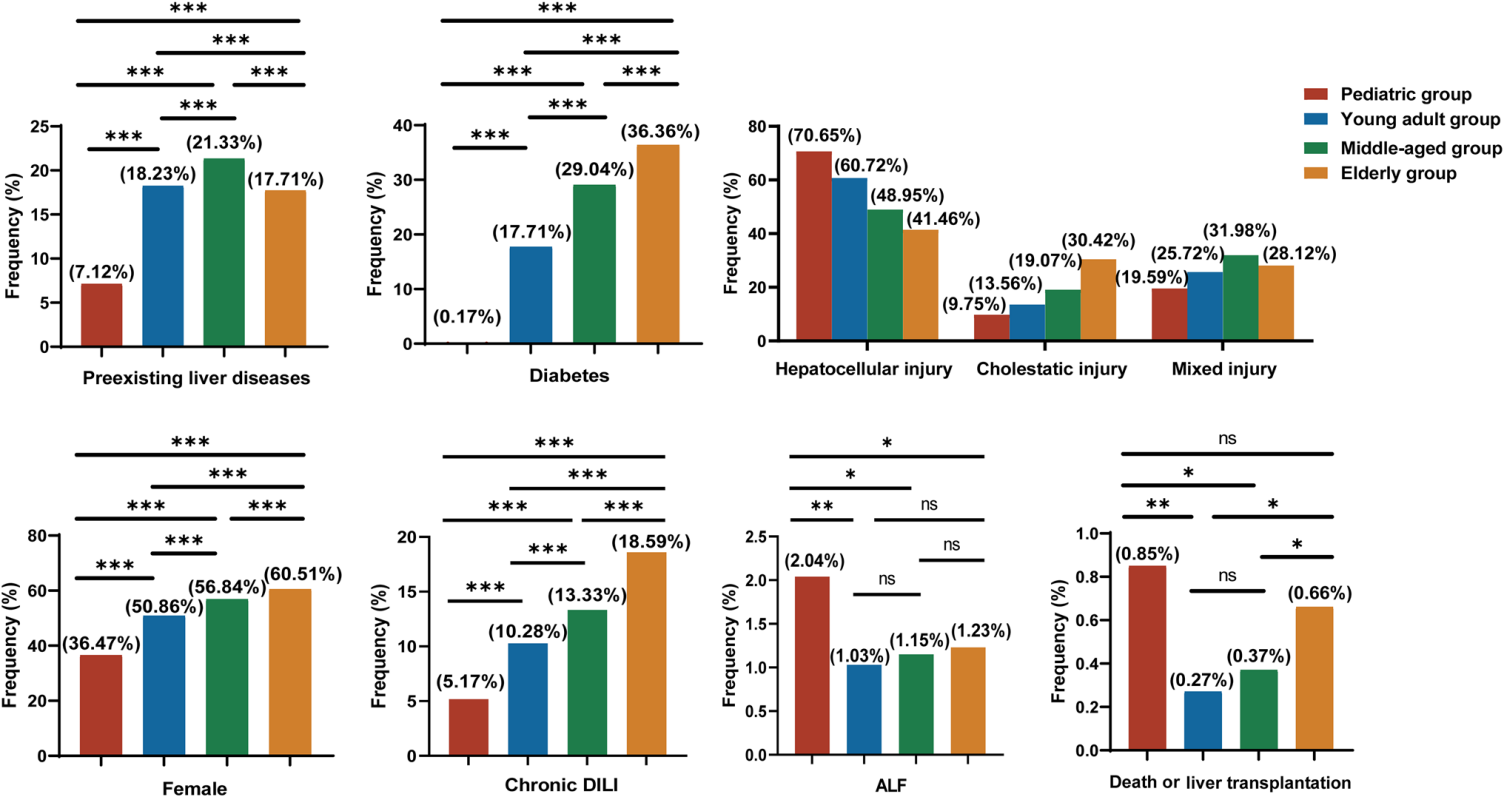
Clinical features of the different age groups. Note, *p < 0.05; **p < 0.01; ***p < 0.001; ns, not significant

The sex, preexisting liver disease status, clinical type, and prognosis of patients with DILI were compared according to age. As shown in Fig. 2, the proportion of females increased with age from 36.47% in the pediatric group to 60.51% in the elderly group. Notably, male predominance was observed only in the pediatric group. Patients in the middle-aged group (21.33%) had a significantly greater proportion of preexisting liver disease than did those in the other age groups (*p* < 0.001). Additionally, the elderly group showed a significantly greater proportion of diabetes (36.36%) than other age groups (*p* < 0.001). In contrast, there were minimal instances of preexisting liver disease (7.12%) and diabetes (0.17%) among children. There were significant differences in the clinical types of DILI among the different age groups (*p* < 0.001). In the pediatric group, hepatocellular DILI was more prevalent (70.65%), whereas in the elderly group, cholestatic DILI was more common (30.42%). With advancing age, the proportion of hepatocellular DILI gradually decreased, while the proportion of cholestatic DILI increased. Notably, the chronic DILI incidence increased with age. Accordingly, compared with that in young patients, DILI in elderly patients was more frequently chronic (18.59%, *p* < 0.001). There was a relationship between ALF and fatal outcomes (death or liver transplantation), and both features were more prevalent in the pediatric and elderly groups, especially in the pediatric group (2.04%, *p* = 0.041, and 0.85%, *p* = 0.007, respectively) (Fig. 2) (Supplementary Table 5).

## Etiology of DILI

**Fig. 3:**
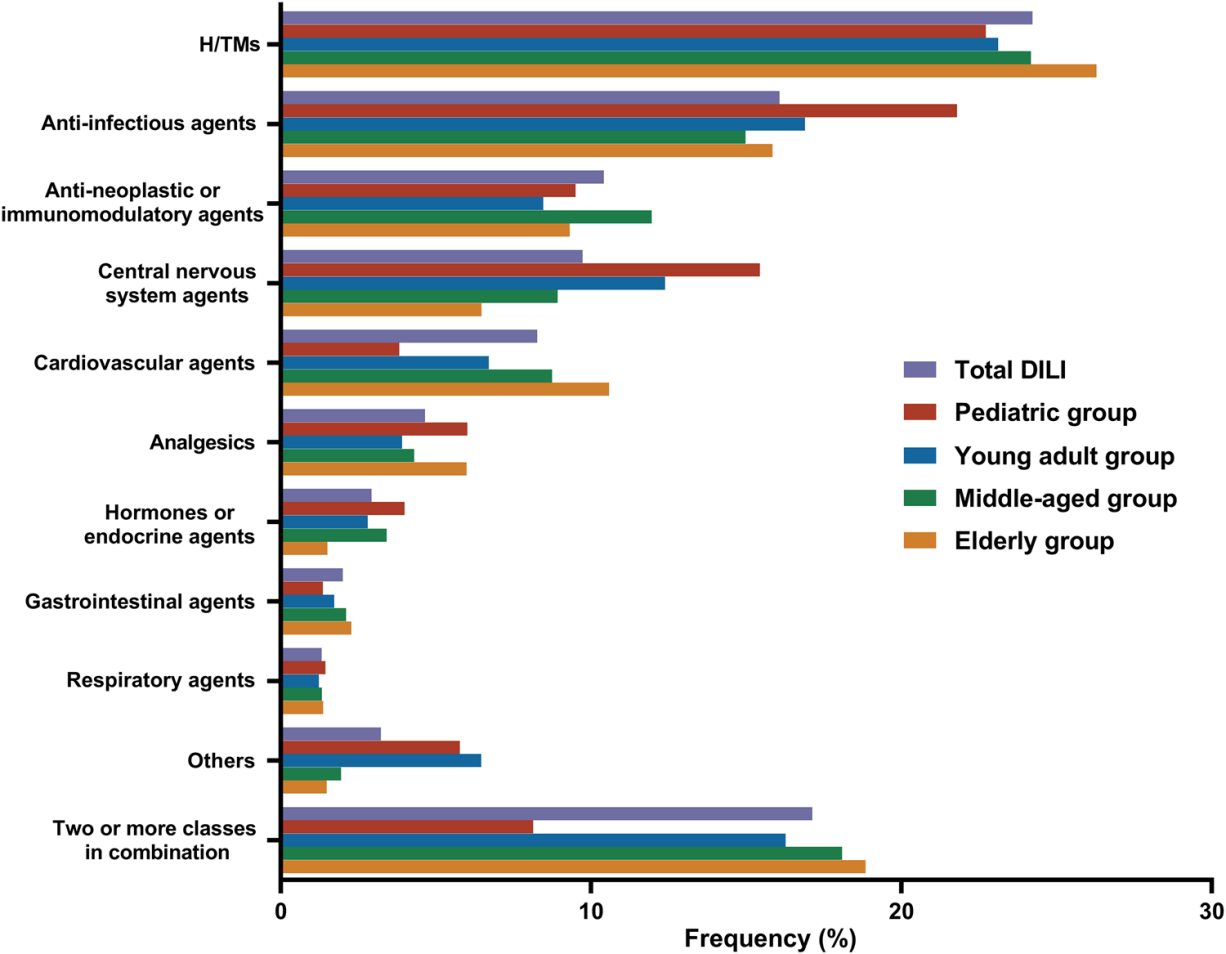
The distribution of DILI etiologies in different age groups

As depicted in Fig. 3, the implicated drugs were classified based on their respective classes and primary clinical indications. The majority of DILI events were attributed to drugs within single classes. Herbal dietary supplements or traditional medicines (H/TMs) (24.23%), anti-infectious agents (16.08%), and antineoplastic or immunomodulatory agents (10.41%) were among the top three classes of implicated agents, followed by central nervous system agents (9.73%), analgesics (4.65%), hormones or endocrine agents (2.93%), gastrointestinal agents (2.00%), and respiratory agents (1.32%) (Supplementary Table 6). Notably, among the patients with DILI caused by anti-infectious agents, 35.77% (1032 out of 2885) of cases were specifically attributed to antituberculosis agents, including isoniazid, rifampicin, pyrazinamide, and ethambutol (Supplementary Table 7). Beyond single-agent involvement, implicated agents spanned two or more classes in 15.75% of patients with DILI (Fig. 3) (Supplementary Table 6).

For all age groups, H/TMs, anti-infectious agents, and antineoplastic or immunomodulatory agents were the most commonly implicated causative agents. Additionally, central nervous system agents and anti-infectious agents were the primary causative agents in the pediatric and young adult groups, whereas cardiovascular agents and H/TMs predominated in the middle-aged and elderly groups. In the pediatric group, DILI was mostly caused by drugs within single classes, while the tendency for combinations of two or more agents increased with age (Fig. 3) (Supplementary Table 6).

## Trends in the incidence of DILI

**Fig. 4:**
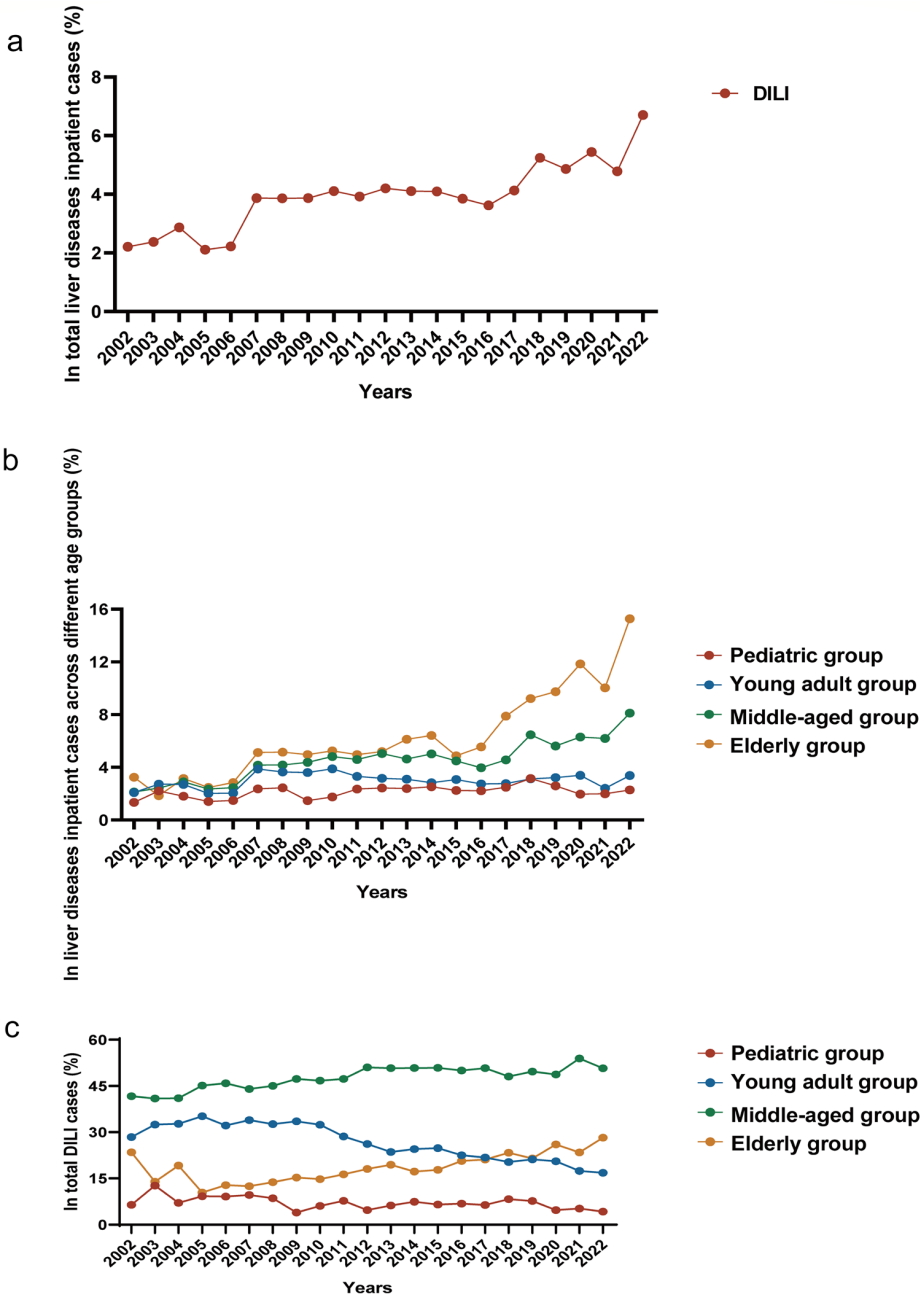
Trends in the incidence of DILI between 2002 and 2022. **a** Trends in the incidence of DILI among inpatients with total liver disease; **b** trends in the incidence of DILI in different age groups; **c** trends in the distribution of DILI patients in various age groups

From January 1, 2002, to December 31, 2022, a total of 487,018 patients with liver disease were hospitalized at the Fifth Medical Center of the PLA General Hospital in Beijing, China, with 19,628 patients diagnosed with DILI. As illustrated in Fig. 4a, the incidence of DILI among patients with liver disease exhibited an upward trend, increasing from 2.21% in 2002 to 6.70% in 2022 (Supplementary Table 8). Furthermore, there was an overall upward trend in the proportion of DILI among patients with liver diseases across different age groups, with the highest incidence observed in the elderly group (Fig. 4b) (Supplementary Table 9). Intriguingly, within the population of DILI patients, there was a yearly upward trend in the proportion of elderly individuals with DILI (Fig. 4c) (Supplementary Table 10). These results indicated that DILI is increasingly prevalent among hospitalized patients, and the age of onset is increasing.

## Discussion

This is the first large-scale retrospective study on DILI among hospitalized patients across various age subgroups in mainland China. In our single-center study, examining the case records of nearly half a million hospitalized patients with liver disease, 4.03% of the patients received a diagnosis of DILI during the period from January 1, 2002, to December 31, 2022. Our findings suggest an upward trend in the incidence rate of DILI among hospitalized patients with liver diseases over the past 20 years, increasing from 2.21% in 2002 to 6.70% in 2022. This trend was particularly noteworthy in the middle-aged and elderly populations and deserves greater attention (Fig. 4).

We observed significant age-related differences in DILI. Across all age groups, the middle-aged group had the highest incidence of DILI, while the pediatric group had the lowest incidence, consistent with findings from previous surveys based primarily on hospitalization medical records[8, 16, 17]. The lower incidence of DILI among children may be attributed to several factors. Children typically receive fewer medications than adults do, and they are less likely to consume alcohol or smoke—both of which can impact drug metabolism[18]. Notably, the incidence of DILI among middle-aged and elderly people is increasing annually. With the aging of the population, the burden of DILI is also expected to increase.

Compared with male patients, female patients have been reported to be at greater risk of DILI according to various published retrospective and prospective DILI studies[5, 19–22]. However, in our study, while Female constituted a slightly larger proportion of the overall DILI population, a greater number of males were observed in the pediatric group. Notably, the sex-associated risk of DILI was not evident in the clinical outcomes. These results suggest that female sex may not be a specific risk factor for the occurrence of DILI, especially in children.

Currently, antimicrobial agents and acetaminophen are the leading causes of DILI in Western countries, while H/TMs have emerged as the principal contributors to DILI in Asian populations [23, 24]. Our data indicate that H/TMs, anti-infectious agents, and antineoplastic or immunomodulatory agents constitute the major implicated causative agents of DILI. In China, H/TMs boast a rich history of use and are extensively employed for the prevention and treatment of various diseases. Some individuals may erroneously assume that H/TM products are inherently safe due to their natural origins [25]. However, the actual ingredients in many herbal products currently in circulation are intricate and often unclear, especially in formulations with multiple compounds. Owing to a lack of stringent standards and supervision, herbs may be procured through the substitution of alternative plant species. These substitutes can be intentionally or inadvertently adulterated with heavy metals, herbicides, pesticides, or microorganisms, posing potential risks of hepatotoxicity [26]. Consequently, unlike those of conventional drugs, the safety profiles of H/TMs are not always well defined. In addition, more than 25% of DILI cases were attributed to anti-infectious agents, antineoplastic or immunomodulatory agents and H/TMs. China is among the countries with the highest global burden of cancer, and a substantial portion of individuals receiving cancer treatment are exposed to antineoplastic or immunomodulatory agents, thereby facing potential risks of hepatotoxicity [27, 28]. Moreover, according to a survey, approximately two-thirds of inpatients in China were prescribed antibiotics, particularly tuberculosis patients [29]. Antibiotic overuse has emerged as a major concern in China and is associated with an increased risk of liver injury in these situations.

Interestingly, our findings suggest that age may influence susceptibility to specific drugs, leading to DILI. Among the drugs commonly implicated in DILI, central nervous system agents were more prevalent in pediatric patients, while cardiovascular agents were more commonly observed in elderly patients. Central nervous system agents were implicated in 15.44% of the DILI cases among children, with the majority being anticonvulsants prescribed for seizures or psychiatric issues. A previous study revealed a greater frequency of hepatotoxicity associated with antiepileptic drugs, such as valproic acid, in children. Among them, children aged 6 and younger, as well as those undergoing treatment with multiple antiepileptic drugs, appear to be at increased risk [30, 31].

With advancing age, individuals are more susceptible to developing various chronic illnesses. Compared to other age groups, elderly individuals require a greater number of medications, encompassing cardiovascular, gastrointestinal, and respiratory agents, among others. Our study revealed a considerable increase in the proportion of cardiovascular agents associated with DILI among elderly individuals, which was distinct from the findings in other age groups. Furthermore, approximately 19% of the elderly patients used a combination of at least two classes of prescription medications, with the majority involving concurrent usage of H/TMs. A previous study revealed that approximately 88% of elderly patients utilized at least one prescription medication, 36% of whom were prescribed five or more prescription medications [32]. A report from the US Food and Drug Administration assessing serious adverse drug events between 1998 and 2005, indicated that although elderly patients constituted only 12.6% of the total US population, they contributed 33.6% of adverse drug events—a proportion substantially higher than anticipated[33]. In addition, while a young liver exhibits remarkable regenerative capacity, the hepatic reserve diminishes with age, especially after the patient reaches 60 years, where it exerts dynamic effects on drug concentrations in liver tissue [34–36]. In summary, concerning DILI, polypharmacy in elderly individuals increases the risk due to both the quantity of prescribed medications and unforeseen drug‒drug interactions. Consequently, DILI associated with polypharmacy emerges as a notable concern in the elderly population.

In this study, cholestatic DILI appeared to be more prevalent among elderly individuals, while younger individuals were more likely to present with hepatocellular DILI. However, the biological basis for this phenotypic difference remains unclear. The greater incidence of cholestatic DILI among elderly individuals may be associated with age-related declines in biliary function and liver regeneration [12]. An analysis of the World Health Organization database covering 236 drugs linked to DILI revealed that lipophilic medications may exhibit increased absorption in elderly patients [13]. Additionally, drugs influencing biliary excretion and causing the inhibition of bile salt export pumps may contribute to the observed cholestatic type in elderly patients with DILI [13, 36]. Indeed, while pharmacokinetic alterations exhibit age dependency, the specific ways in which these changes, along with other age-related factors, contribute to this shift are presently unknown. Further research is imperative to elucidate the heightened risk of cholestatic DILI in elderly patients and to establish biomarkers for cholestatic DILI.

Our study revealed a close association between cholestatic DILI and progression to chronic DILI, as well as more adverse outcomes, including ALF and mortality. However, the mechanisms underlying this association remain unclear. Some studies speculate that this association may share similarities with mechanisms involved in other immune-mediated cholestatic liver diseases, such as primary biliary cholangitis [37]. Specifically, these mechanisms involve immune-mediated attacks on the Hering ducts and small bile ducts, disrupting the normal relationship between liver cells and bile ducts and ultimately leading to subsequent adverse outcomes [38, 39]. While there is currently insufficient evidence to definitively establish a correlation between preexisting liver diseases and diabetes with the risk of DILI, it is noteworthy that individuals with preexisting liver diseases or diabetes appear to be more susceptible to severe outcomes following DILI. Our study indicated that both preexisting liver diseases and diabetes were associated with increased chronicity in cases of DILI. Furthermore, preexisting liver diseases were correlated with severe adverse outcomes (ALF and mortality or the need for liver transplantation).

Elderly individuals often exhibit a higher prevalence of underlying diseases, increased medication usage, and a reduced capacity for liver detoxification and elimination. As previously mentioned, when DILI occurs, elderly patients are more susceptible to developing cholestatic DILI and chronic DILI, potentially increasing the risk of liver failure and mortality in this population.

Notably, despite children having minimal underlying disease, using fewer medications, and facing a lower risk of DILI than adults, children with DILI had an increased incidence of ALF, even requiring liver transplantation or even resulting in mortality in our study. In a multicenter study, DILI in children was shown to account for up to 20% of cases of pediatric ALF necessitating liver transplant surgery[40]. The mortality rates in reported studies on DILI among children varied from 4 to 31% [30, 41]. These findings demonstrate the potential severity of DILI in children. However, the extent to which most drugs cause DILI in children to differ from that in adults remains largely unknown. The mechanistic data available predominantly focus on DILI in adults, with limited information on pediatric patients. The absence of regulatory guidance specific to children with DILI may stem from insufficient data. However, further studies are needed to enhance our understanding of the likelihood of DILI in children, pinpoint risk factors, and ensure the safe administration of potentially hepatotoxic drugs in pediatric populations.

In summary, the prevention and treatment of DILI currently face major challenges. As China’s population ages, the population of middle-aged and elderly individuals with multiple chronic conditions and the concurrent use of various medications is substantial. Widespread noncompliance and irrational drug use, coupled with a lack of familiarity among non-hepatology clinicians in diagnosing and managing DILI and imperfect risk management measures by pharmaceutical companies post-market approval, contribute to the escalating incidence of DILI. In response, our hospital has implemented preventive measures for DILI. Patient information and data on medications suspected to cause DILI are synchronously uploaded to the China National Adverse Drug Reaction (ADR) Monitoring Center. This center regularly publishes ADR reporting data for clinicians and the public to stay informed [42]. Clinical pharmacists are integrated into the treatment decision-making team to ensure the rationality and safety of clinical drug use. For high-risk drugs such as vancomycin and lamotrigine characterized by a narrow safety window, standardized blood drug concentration monitoring is conducted to reduce toxicity resulting from irrational drug use. Despite these efforts, there remains a substantial unmet clinical need. Regulatory authorities need to scientifically address potentially hepatotoxic drugs by implementing reasonable measures, such as suspending production or sales, withdrawing drugs from the market, revising drug labels, and restricting usage. Pharmaceutical companies need to establish comprehensive risk management measures, including the creation of drug safety alert departments, formulation of appropriate monitoring and risk management strategies, proactive research, label revisions, and risk communication. Clinicians need to incorporate risk management measures for DILI into clinical practice, such as regular monitoring during treatment, timely identification of suspected DILI, clear diagnosis, and discontinuing or reducing dosages.

Considering age-related pharmacokinetic changes, bile stasis, and the widespread use of multiple medications, elderly individuals face a higher risk of DILI. Additionally, elderly people are more prone to chronic DILI and ALF. However, liver transplantation options are limited for elderly individuals with DILI-induced liver failure. Therefore, clinicians should recognize the heightened risk of adverse outcomes in elderly people following DILI and exercise increased caution when prescribing drugs. Adjusting drug doses, discontinuing unnecessary medications, and conducting detailed and frequent medication reviews and regular liver function tests may help mitigate this risk. Furthermore, certain drugs may metabolize differently in children than in adults. Clinicians should be vigilant about the potential for severe liver damage caused by commonly used anti-infective or central nervous system drugs in children. Drug doses need to be adjusted based on age and weight, with a thorough assessment of the risk–benefit ratio. Similar to elderly individuals, children require regular monitoring of liver function when using potentially hepatotoxic drugs.

This study has several limitations. First, this was a retrospective, single-center study, implying that the data may not represent all regions and levels in China. Second, the absence of defined hepatic injury thresholds for newborns and children necessitated our reliance on adult DILI standards, potentially introducing deviations in the screening results. Throughout this study, our focus was on DILI among hospitalized patients, leading to a deficiency in research on outpatients. Consequently, it is possible that our study included more severe cases of DILI than those typically encountered in the general population.

## Conclusions

This study explored age-related variations in DILI and provides evidence for future investigations into age-specific DILI. The heightened risk of adverse outcomes following DILI in children and elderly people underscores the necessity of strengthening the monitoring and reporting of DILI cases in these age groups. Future efforts may involve international, multicenter collaborations to enhance our understanding of the risk factors for DILI across different age groups.

### Supplementary Information

Below is the link to the electronic supplementary material.

Supplementary file1 (DOCX 229 KB)

## Data Availability

No additional data are available.

## Abbreviations

DILI: Drug-induced liver injury
PLA: People’s Liberation Army
H/TMs: Herbal dietary supplements or traditional medicines
ALF: Acute liver failure
RUCAM: Roussel-Uclaf causality assessment method
ULN: Upper limit of normal
SPSS: Statistical Package for the Social Sciences
