# Supplementary material for: Age-related differences in drug-induced liver injury: a retrospective single-center study from a large liver disease specialty hospital in China, 2002–2022
Source: Hepatol Int. 2024 Jun 19;18(4):1202–13. doi: 10.1007/s12072-024-10679-1 (PMC11297843; doi:10.1007/s12072-024-10679-1)
Supplement: Supplementary file 1 — Supplementary file1 (DOCX 229 KB) [file 12072_2024_10679_MOESM1_ESM.docx]

**Supplementary Material**


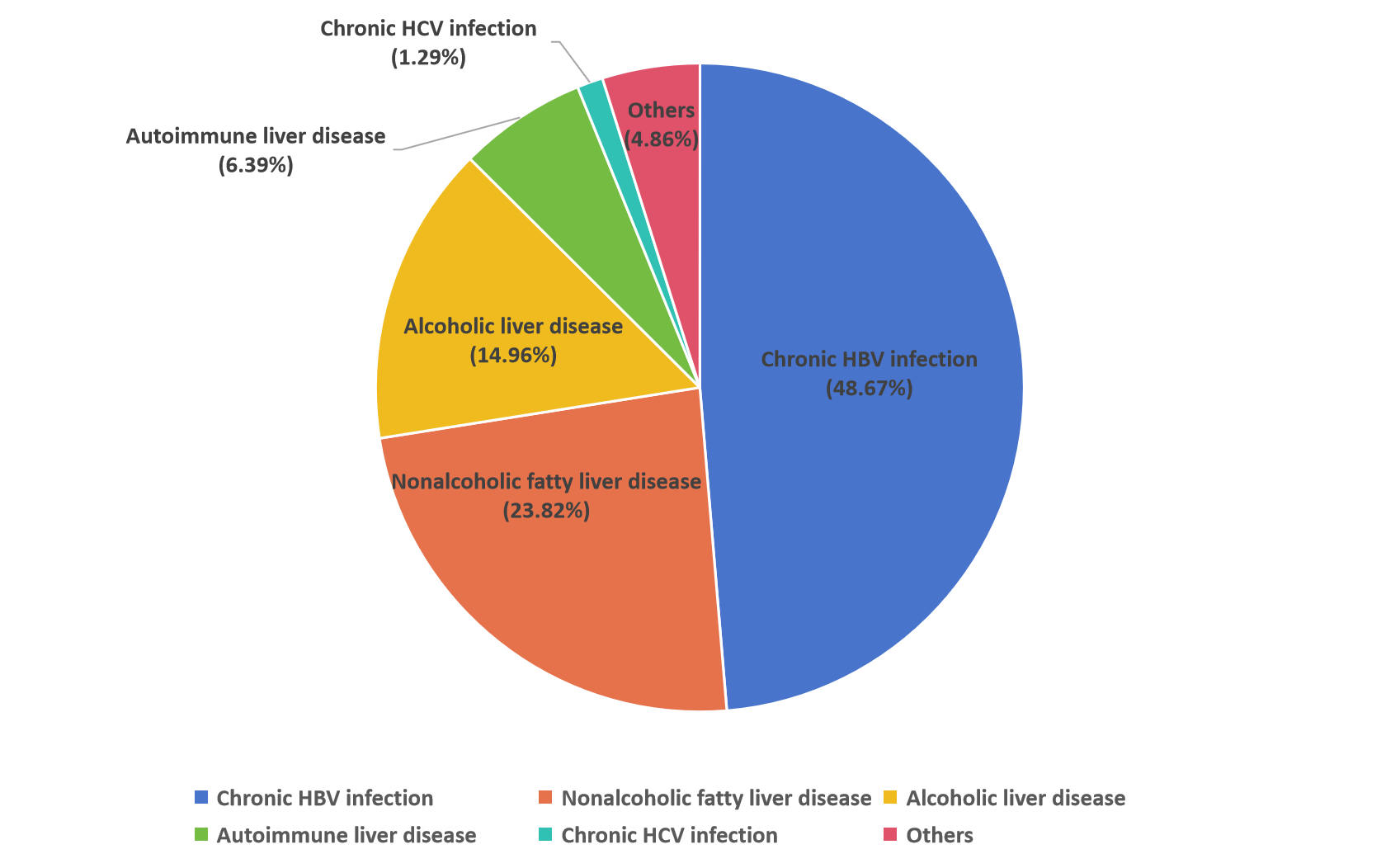


Supplementary Figure 1. The distribution of DILI patients with preexisting liver disease in the study.

Supplementary Table 1. Comparison of DILI prognosis according to different clinical types

|  | Cholestatic type (n=3465) | Mixed type (n=5179) | Hepatocellular type (n=9302) | *P* value |
| --- | --- | --- | --- | --- |
| Chronic DILI (n, %) | 760 (21.93) | 643 (12.42) | 942 (10.13) | <0.001 |
| Death or liver transplantation (n, %) | 28 (0.81) | 19 (0.37) | 31 (0.33) | 0.001 |
| Progression to ALF (n, %) | 66 (1.90) | 58 (1.12) | 90 (0.97) | <0.001 |

Supplementary Table 2. Comparison of DILI prognosis between patients with and without preexisting liver disease

|  | DILI with preexisting liver disease (n=3695) | DILI without preexisting liver disease (n=14251) | *P* value |
| --- | --- | --- | --- |
| Chronic DILI (n, %) | 998 (27.01) | 1347 (9.45) | <0.001 |
| Death or liver transplantation (n, %) | 27 (0.73) | 51 (0.36) | 0.002 |
| Progression to ALF (n, %) | 66 (1.79) | 148 (1.04) | <0.001 |

Supplementary Table 3. Comparison of DILI prognosis between patients with and without diabetes

|  | DILI with diabetes (n=4623) | DILI without diabetes (n=13323) | *P* value |
| --- | --- | --- | --- |
| Chronic DILI (n, %) | 886 (19.17) | 1459 (10.95) | <0.001 |
| Death or liver transplantation (n, %) | 25 (0.54) | 53 (0.40) | 0.200 |
| Progression to ALF (n, %) | 63 (1.32) | 151 (1.15) | 0.216 |

Supplementary Table 4. Comparison of DILI prognosis between males and females

|  | Males (n=8123) | Females (n=9823) | *P* value |
| --- | --- | --- | --- |
| Chronic DILI (n, %) | 1044 (12.85) | 1301 (13.24) | 0.438 |
| Death or liver transplantation (n, %) | 33 (0.41) | 45 (0.46) | 0.599 |
| Progression to ALF (n, %) | 87 (10.71) | 127 (12.93) | 0.173 |

Supplementary Table 5. Clinical features of the different age groups

|  | Pediatric group (n=1179) | Young adult group (n=4455) | Middle-aged group (n=8805) | Elderly group (n=3507) | *P* value |
| --- | --- | --- | --- | --- | --- |
| Females (n, %) | 430 (36.47) | 2266 (50.86) | 5005 (56.84) | 2122 (60.51) | <0.001 |
| Preexisting liver diseases (n, %) | 84 (7.12) | 812 (18.23) | 1878 (21.33) | 921 (17.71) | <0.001 |
| Diabetes (n, %) | 2 (0.17) | 789 (17.71) | 2557 (29.04) | 1275 (36.36) | <0.001 |
| Chronic DILI (n, %) | 61 (5.17) | 458 (10.28) | 1174 (13.33) | 652 (18.59) | <0.001 |
| Progression to ALF (n, %) | 24 (2.04) | 46 (1.03) | 101 (1.15) | 43 (1.23) | 0.041 |
| Death or liver transplantation (n, %) | 10 (0.85) | 12 (0.27) | 33 (0.37) | 23 (0.66) | 0.007 |
| **Clinical types of DILI (n, %)** |  |  |  |  | <0.001 |
| Hepatocellular type | 833 (70.65) | 2705 (60.72) | 4310 (48.95) | 1454 (41.46) |  |
| Cholestatic type | 115 (9.75) | 604 (13.56) | 1679 (19.07) | 1067 (30.42) |  |
| Mixed type | 231 (19.59) | 1146 (25.72) | 2816 (31.98) | 986 (28.12) |  |

Supplementary Table 6. Implicated agents across various age groups

| Medications | Total DILI (n, %) | Pediatric group (n, %) | Young adult group (n, %) | Middle-aged group (n, %) | Elderly group (n, %) |
| --- | --- | --- | --- | --- | --- |
| H/TMs | 4349 (24.23) | 268 (22.73) | 1030 (23.12) | 2129 (24.18) | 922 (26.29) |
| Anti-infectious agents | 2885 (16.08) | 257 (21.80) | 753 (16.90) | 1319 (14.98) | 556 (15.85) |
| Antineoplastic or immunomodulatory agents | 1869 (10.41) | 112 (9.50) | 377 (8.46) | 1053 (11.96) | 327 (9.32) |
| Central nervous system agents | 1747 (9.73) | 182 (15.44) | 552 (12.39) | 786 (8.93) | 227 (6.47) |
| Cardiovascular agents | 1485 (8.27) | 45 (3.82) | 299 (6.71) | 770 (8.75) | 371 (10.58) |
| Analgesics | 834 (4.65) | 71 (6.02) | 174 (3.91) | 379 (4.30) | 210 (5.99) |
| Hormones or endocrine agents | 526 (2.93) | 47 (3.99) | 125 (2.81) | 301 (3.42) | 53 (1.51) |
| Gastrointestinal agents | 359 (2.00) | 16 (1.36) | 77 (1.73) | 186 (2.11) | 80 (2.28) |
| Respiratory agents | 237 (1.32) | 17 (1.44) | 55 (1.23) | 117 (1.33) | 48 (1.37) |
| Others | 580 (3.23) | 68 (5.77) | 288 (6.46) | 172 (1.95) | 52 (1.48) |
| Two or more classes in combination | 3075 (17.13) | 96 (8.14) | 725 (16.27) | 1593 (18.09) | 661 (18.85) |

Supplementary Table 7. Distribution of DILI etiologies

| Classes of implicated agents | N | Causal agents (n>10) |
| --- | --- | --- |
| H/TMs | 4349 | Rhei Radix et Rhizoma (712), polygoni multiflori radix (435), epimedii folium (389), weight-loss pills (359), psoraleae fructus (249), greater celandine (243), health or sexual enhancement products (234), folium sennae (167), ephedrae herba (119), albumen powder (108), dictamni cortex (84), radix stephaniae epigaeae (77), turmeric (65), senecionis scandentis hebra (46), viaminate (36), fructus xanthii (33), cinnabar (29), bupleurum Chinense (21), rhizoma dioscoreae bulbiferae (17), tripterygii radix (14), calcium (11), bruceae fructus (11), etc. (890) |
| Anti-infectious agents | 2885 | Rifampicin (317), isoniazid (282), amoxicillin-clavulanate (253), pyrazinamide (248), levofloxacin (213), minocycline (195), [ethambutol](https://www.sciencedirect.com/topics/medicine-and-dentistry/ethambutol) (185), moxifloxacin (177), voriconazole (155), azithromycin (147), fluconazole (125), vancomycin (103), piperacillin (73), etronidazole (67), ornidazole (51), sulbenicillin (42), flucloxacillin (31), aztreonam (25), cefotiam (22), cefoperazone (20), meropenem (18), teicoplanin (17), clindamycin (17), ketoconazole (16), lamivudine (12), etc.(74) |
| Antineoplastic or immunomodulatory agents | 1869 | Mercaptopurine (332), gemtuzumab (253), tioguanine (228), methotrexate (189), basiliximab (167), daunorubicin (136), crizotinib (104), pemetrexed (87), gefitinib (71), sorafenib (65), pegaspargase (55), dasatinib (42), irinotecan (33), cytarabine (24), mitoxantrone (12), etc.(71) |
| Central nervous system agents | 1747 | Valproic acid (356), carbamazepine (265), lamotrigine (234), phenytoin (216), paroxetine (182), diazepam (142), olanzapine (91), flupenthixol (73), melitracen (62), gabapentin (35), escitalopram (29), alprazolam (15), clonazepam (11) , etc.(36) |
| Cardiovascular agents | 1485 | Atorvastatin (609), simvastatin (315), edaravone (211), digoxin (71), amlodipine (62), hemocoagulase (54), granulocyte colony-stimulating factor (21), ramipril (19), rosuvastatin (14), mevastatin (13), etc.(96) |
| Analgesics | 834 | Acetaminophen (415), diclofenac (87), meloxicam (75), ibuprofen (63), celecoxib (44), ketoprofen (33), fentanyl (21), dulcetin (13), etc.(83) |
| Hormones or endocrine agents | 526 | leflunomide (61), hydrocortisone (53), methylprednisolone (48), metformin (45), cyclophosphamide (42), propylthiouracil (38), cyclosporine (37), colchicine (34), tacrolimus (30), dexamethasone (26), progestin (22), azathioprine (18), allopurinol (16), methimazole (14), levothyroxine sodium (13), insulin (12), etc.(17) |
| Gastrointestinal agents | 359 | Omeprazole (71), lansoprazole (59), sulfasalazine (48), rabeprazole (39), mesalazine (36), diammonium glycyrrhizinate (27), pantoprazole (19), ranitidine (14), esomeprazole (12), cimetidine (11), etc.(23) |
| Respiratory agents | 237 | Theophylline (63), oseltamivir (55), dosophylline (31), Ambroxol (26), dioxopromazine (14), etc.(48) |
| Others | 580 | Toxic food (203), Hair dye (109), paint (85), pesticide (12), etc. (171) |
| Two or more classes in combination | 3075 | Combinations of medications that work on different systems (881), or of medications and H/TM (2194) |

Supplementary Table 8. Proportion of DILI patients in the total number of liver disease inpatients from January 1, 2002, to December 31, 2022, at the Fifth Medical Center of the PLA General Hospital, Beijing, China

| Year | Total number of liver disease inpatients | Number of DILI patients | Percentage of DILI patients in liver disease inpatients (%) | 95% CI |
| --- | --- | --- | --- | --- |
| 2002 | 11,946 | 264 | 2.21 | [1.89-2.52] |
| 2003 | 9958 | 237 | 2.38 | [2.01-2.75] |
| 2004 | 11,812 | 339 | 2.87 | [2.53-3.22] |
| 2005 | 19,526 | 412 | 2.11 | [1.88-2.33] |
| 2006 | 24,640 | 547 | 2.22 | [2.02-2.42] |
| 2007 | 16,382 | 634 | 3.87 | [3.55-4.18] |
| 2008 | 20,311 | 784 | 3.86 | [3.58-4.14] |
| 2009 | 23,669 | 916 | 3.87 | [3.61-4.13] |
| 2010 | 21,922 | 901 | 4.11 | [3.84-4.39] |
| 2011 | 29,031 | 1138 | 3.92 | [3.68-4.15] |
| 2012 | 29,119 | 1223 | 4.20 | [3.96-4.44] |
| 2013 | 31,192 | 1282 | 4.11 | [3.88-4.33] |
| 2014 | 32,146 | 1318 | 4.10 | [3.88-4.32] |
| 2015 | 34,753 | 1338 | 3.85 | [3.64-4.06] |
| 2016 | 34,751 | 1258 | 3.62 | [3.42-3.82] |
| 2017 | 30,823 | 1273 | 4.13 | [3.90-4.36] |
| 2018 | 21,870 | 1146 | 5.24 | [4.93-5.54] |
| 2019 | 22,325 | 1085 | 4.86 | [4.57-5.16] |
| 2020 | 12,077 | 657 | 5.44 | [5.01-5.87] |
| 2021 | 20,377 | 974 | 4.78 | [4.48-5.09] |
| 2022 | 28,388 | 1902 | 6.70 | [6.41-7.00] |

Supplementary Table 9. Proportion of DILI patients in the total number of liver disease inpatients across various age groups from January 1, 2002, to December 31, 2022, at the Fifth Medical Center of the PLA General Hospital, Beijing, China

| Year | Pediatric group | | Young adult group | | Middle-aged group | | Elderly group | |
| --- | --- | --- | --- | --- | --- | --- | --- | --- |
|  | The number of liver disease inpatients | The number of DILI patients (n, %) | The number of liver disease inpatients | The number of DILI patients (n, %) | The number of liver disease inpatients | The number of DILI patients (n, %) | The number of liver disease inpatients | The number of DILI patients (n, %) |
| 2002 | 1269 | 17 (1.34) | 3594 | 75 (2.09) | 5177 | 110 (2.12) | 1906 | 62 (3.25) |
| 2003 | 1361 | 30 (2.20) | 2835 | 77 (2.72) | 3976 | 97 (2.44) | 1786 | 33 (1.85) |
| 2004 | 1336 | 24 (1.80) | 4118 | 111 (2.70) | 4793 | 139 (2.90) | 1565 | 65 (4.15) |
| 2005 | 2701 | 38 (1.41) | 7172 | 145 (2.02) | 7915 | 186 (2.35) | 1738 | 43 (2.47) |
| 2006 | 3352 | 50 (1.49) | 8628 | 176 (2.04) | 10206 | 251 (2.46) | 2454 | 70 (2.85) |
| 2007 | 2576 | 61 (2.37) | 5556 | 215 (3.87) | 6709 | 279 (4.16) | 1541 | 79 (5.13) |
| 2008 | 2736 | 67 (2.45) | 7032 | 256 (3.64) | 8445 | 353 (4.18) | 2098 | 108 (5.15) |
| 2009 | 2430 | 36 (1.48) | 8533 | 307 (3.60) | 9889 | 433 (4.38) | 2817 | 140 (4.97) |
| 2010 | 3138 | 55 (1.75) | 7505 | 292 (3.89) | 8743 | 421 (4.82) | 2536 | 133 (5.24) |
| 2011 | 3745 | 88 (2.35) | 9816 | 326 (3.32) | 11724 | 538 (4.59) | 3746 | 186 (4.96) |
| 2012 | 2381 | 58 (2.44) | 10119 | 320 (3.16) | 12357 | 624 (5.05) | 4262 | 221 (5.19) |
| 2013 | 3346 | 80 (2.39) | 9748 | 302 (3.10) | 14039 | 651 (4.64) | 4059 | 249 (6.13) |
| 2014 | 3890 | 98 (2.52) | 11378 | 323 (2.84) | 13341 | 670 (5.02) | 3537 | 227 (6.42) |
| 2015 | 3860 | 87 (2.25) | 10823 | 332 (3.07) | 15188 | 681 (4.48) | 4882 | 238 (4.88) |
| 2016 | 3876 | 86 (2.22) | 10318 | 283 (2.74) | 15876 | 629 (3.96) | 4681 | 260 (5.55) |
| 2017 | 3261 | 81 (2.48) | 10007 | 277 (2.77) | 14142 | 646 (4.57) | 3413 | 269 (7.88) |
| 2018 | 3013 | 95 (3.15) | 7447 | 233 (3.13) | 8515 | 551 (6.47) | 2895 | 267 (9.22) |
| 2019 | 3208 | 83 (2.59) | 7133 | 230 (3.22) | 9591 | 539 (5.62) | 2393 | 233 (9.74) |
| 2020 | 1570 | 31 (1.97) | 3982 | 135 (3.39) | 5082 | 320 (6.30) | 1443 | 171 (11.85) |
| 2021 | 2567 | 51 (1.99) | 7056 | 170 (2.41) | 8483 | 525 (6.19) | 2271 | 228 (10.04) |
| 2022 | 3494 | 80 (2.29) | 9476 | 320 (3.38) | 11903 | 965 (8.11) | 3515 | 537 (15.28) |

Supplementary Table 10. Proportion of DILI patients in different age groups in the total number of DILI patients from 2002 to 2022 at the Fifth Medical Center of the PLA General Hospital, Beijing, China (n, %)

| Year | The total number of DILI patients | Pediatric group (n, %) | Young adult group (n, %) | Middle-aged group (n, %) | Elderly group (n, %) |
| --- | --- | --- | --- | --- | --- |
| 2002 | 264 | 17 (6.44) | 75 (28.41) | 110 (41.67) | 62 (23.48) |
| 2003 | 237 | 30 (12.66) | 77 (32.49) | 97 (40.93) | 33 (13.92) |
| 2004 | 339 | 24 (7.08) | 111 (32.74) | 139 (41.00) | 65 (19.17) |
| 2005 | 412 | 38 (9.22) | 145 (35.19) | 186 (45.15) | 43 (10.44) |
| 2006 | 547 | 50 (9.14) | 176 (32.18) | 251 (45.89) | 70 (12.80) |
| 2007 | 634 | 61 (9.62) | 215 (33.91) | 279 (44.01) | 79 (12.46) |
| 2008 | 784 | 67 (8.55) | 256 (32.65) | 353 (45.03) | 108 (13.78) |
| 2009 | 916 | 36 (3.93) | 307 (33.52) | 433 (47.27) | 140 (15.28) |
| 2010 | 901 | 55 (6.10) | 292 (32.41) | 421 (46.73) | 133 (14.76) |
| 2011 | 1138 | 88 (7.73) | 326 (28.65) | 538 (47.28) | 186 (16.34) |
| 2012 | 1223 | 58 (4.74) | 320 (26.17) | 624 (51.02) | 221 (18.07) |
| 2013 | 1282 | 80 (6.24) | 302 (23.56) | 651 (50.78) | 249 (19.42) |
| 2014 | 1318 | 98 (7.44) | 323 (24.51) | 670 (50.83) | 227 (17.22) |
| 2015 | 1338 | 87 (6.50) | 332 (24.81) | 681 (50.90) | 238 (17.79) |
| 2016 | 1258 | 86 (6.84) | 283 (22.50) | 629 (50.00) | 260 (20.67) |
| 2017 | 1273 | 81 (6.36) | 277 (21.76) | 646 (50.75) | 269 (21.13) |
| 2018 | 1146 | 95 (8.29) | 233 (20.33) | 551 (48.08) | 267 (23.30) |
| 2019 | 1085 | 83 (7.65) | 230 (21.20) | 539 (49.68) | 233 (21.47) |
| 2020 | 657 | 31 (4.72) | 135 (20.55) | 320 (48.71) | 171 (26.03) |
| 2021 | 974 | 51 (5.24) | 170 (17.45) | 525 (53.90) | 228 (23.41) |
| 2022 | 1902 | 80 (4.21) | 320 (16.82) | 965 (50.74) | 537 (28.23) |
